# *Mycobacterium bovis* Strains Causing Smear-Positive Human Tuberculosis, Southwest Ireland

**DOI:** 10.3201/eid1412.071135

**Published:** 2008-12

**Authors:** Olabisi Ojo, Stella Sheehan, G. Daniel Corcoran, Melissa Okker, Karen Gover, Vladyslav Nikolayevsky, Timothy Brown, James Dale, Stephen V. Gordon, Francis Drobniewski, Michael B. Prentice

**Affiliations:** University College Cork, Cork, Ireland (O. Ojo, M.B. Prentice); Cork University Hospital, Cork (S. Sheehan, G.D. Corcoran, M.B. Prentice); Veterinary Laboratories Agency Weybridge, Surrey, UK (M. Okker, K. Gover, J. Dale, S.V. Gordon); Health Protection Agency Barts and the London School of Medicine, London, UK (V. Nikolayevsky, T. Brown, F. Drobniewski); 1 Current affiliation: University College Dublin, Dublin, Ireland.

**Keywords:** Mycobacterium bovis, tuberculosis, molecular epidemiology, respiratory tract infections, dispatch

## Abstract

*Mycobacterium bovis* caused 3% of human tuberculosis cases in southwest Ireland during 1998–2006. Of 11 *M. bovis* strains genotyped, 9 belonged to common animal spoligotypes. Seven strains were from sputum and potential sources of human-centered disease transmission. Ten-locus variable-number tandem repeat typing gave unique strain profiles and would detect disease outbreaks.

Bovine tuberculosis occurs worldwide (*1*–*3*). It is caused by *Mycobacterium bovis*, a cattle-adapted member of the *M*. *tuberculosis* complex. *M. bovis* has the broadest host range of pathogenic mycobacteria, infecting domestic and wild mammals, and is classified as a Hazard Group 3 infectious agent (*1*). Human infection follows ingestion of unpasteurized milk or inhalation of droplet nuclei (*1*). In many countries, the risk for *M. bovis* infection in humans has been reduced by a test-and-slaughter program in which infected cattle are identified and culled. This program has eradicated *M. bovis* cattle infection from 11 states of the European Union (*3*). However, the Republic of Ireland and its neighbor the United Kingdom have failed to eradicate bovine tuberculosis (*1*,*4*). In the 1980s, 4%–6% of all cases of laboratory-confirmed tuberculosis in southwest Ireland were caused by *M. bovis* (*5*). Our study results suggest that this remains a problem in Ireland.

Molecular typing systems for pathogenic mycobacteria are important for epidemiologic control because they enable case-linking and outbreak tracing (*6*). We report a molecular epidemiology study that used spoligotyping, mycobacterial interspersed repetitive units–variable-number tandem repeat (MIRU-VNTR) typing, and region of difference (RD) typing of *M. bovis* strains isolated from human residents of Ireland.

## The Study

During 1998–2006, the microbiology laboratory at Cork University Hospital obtained *M*. *tuberculosis* complex isolates from 501 patients (equivalent to 68.5% of notified cases) residing in southwest Ireland (counties Cork and Kerry); 15 were *M. bovis* isolates (3%). Eleven of these *M. bovis* strains were available for testing. Seven additional isolates obtained over this period (from inoculation abscesses) were identified as *M. bovis* BCG and not analyzed. Strains were identified as *M. tuberculosis* complex by using Accuprobe (Gene-Probe, San Diego, CA, USA) and as potential *M. bovis* strains by pyruvate dependence. Definitive identification was performed at the Mycobacterial Reference Unit in London and was based on absence of niacin production and nitrate reductase activity, thiophen-2-carboxylic acid hydrazide negativity, and pyrazinamide resistance (*1*). DNA extraction was performed as described (*2*). DNA controls were *M. tuberculosis* H37Rv and *M. bovis* AF2122/97. A 6-locus panel VNTR (exact tandem repeat [ETR]-A to ETR-F) (*7*) was used initially, then a 10-locus VNTR panel was used (Table 1) (*8*). Spoligotyping was as described by Kamerbeek (*9*). RD analysis was conducted by the method of Brosch et al. (*10*) for RD1, RD4, RD9, RD10, RD12, RDpan, RD17, N-RD17, and N-RD25. This study was reviewed and approved by the Clinical Research Ethics Committee of the Cork Teaching Hospitals (Ref ECM 3).

**Table 1:** Heterogeneity of each MIRU-VNTR locus of *Mycobacterium bovis* isolates, southwest Ireland, 1998–2006*

VNTR locus	Isolate	No. tandem repeat copies												HGDI	Allelic variants
		0	1	2	3	4	5	6	7	8	9	10	11		
1895	QUB1895	0	0	1	2	8	0	0	0	0	0	0	0	0.47	3
2163a	QUB11a	0	0	0	2	0	0	0	0	0	0	4	5	0.69	3
2163b	QUB11b	0	1	0	2	8	0	0	0	0	0	0	0	0.47	3
2165	ETR-A	0	0	0	0	1	2	0	8	0	0	0	0	0.47	3
2461	ETR-B	0	0	0	1	1	8	0	1	0	0	0	0	0.49	4
2687	MIRU24	0	6	5	0	0	0	0	0	0	0	0	0	0.55	2
2996	MIRU26	0	0	0	1	1	7	2	0	0	0	0	0	0.60	4
3232	QUB3232	1	0	0	0	0	0	0	0	7	1	1	1	0.62	5
3336	QUB3336	0	0	1	6	3	0	0	1	0	0	0	0	0.67	4
4052	QUB26	0	0	2	1	8	0	0	0	0	0	0	0	0.47	3

*MIRU, mycobacterial interspersed repetitive units; VNTR, variable-number tandem repeats; HGDI, Hunter Gaston Discriminatory Index; QUB, Queen’s University Belfast; ETR, exact tandem repeat.

Spoligotype signatures were recorded as binary numbers (1 and 0 denoting presence and absence of oligonucleotide spacer, respectively). Signatures were matched against the *M. bovis* spoligotype database (www.mbovis.org) and the global spoligotype database, SpolDB4. Unmatched spoligotypes were sent to the *M. bovis* spoligotype database curator for an authoritative name assignment (SB number). The discriminatory power of VNTR and spoligotyping was calculated by using the Hunter Gaston Discriminatory Index. Two or more spoligotype or VNTR patterns with 100% identity were considered a cluster.

Eight spoligotypes (Table 2) were identified among 11 isolates. Two clusters were identified: 3 strains of SB0140 (ST683) and 2 strains corresponding to SB0139 (ST680) (Table 2). SB0140 (also known as spoligotype A1 or ST1), is the most common spoligotype in animals in Ireland and the United Kingdom (*1**,**11*) (Table 2). SB0139 was previously detected as an isolate from a cow in Northern Ireland in 2000 (SpolDB4; R. Skuce, pers. comm.). Other spoligotypes we identified that had been previously reported in isolates from animals in Ireland were SB0130 (ST691), also described as D1 or ST7; SB0142 (ST679), also described as A2 or ST2*;* and SB1047 (not found in SpolDB4). Three strains were not found in either of the databases. These strains were assigned spoligotypes SB1185, SB1186, and SB1187 through the *M. bovis* spoligotype database. SB1186 and SB1187 differed from the ubiquitous SB140 by absence of spacer 35 and spacers 35 and 37, respectively. Previously reported SB0139, SB0142, and SB1047 differed from SB0140 by absence of spacers 33, 33–34, and 35–36, respectively. SB1185 differed from the established animal strain SB0130 by the absence of spacers 20–21.

**Table 2:** Molecular characteristics of strains and clinical and demographic parameters of 11 patients from whom *Mycobacterium bovis* was isolated, southwest Ireland, 1998–2006*

Study no.	Spoligotype	SpolDB4†	Mbovis. org‡	Other name§	VNTR pattern¶	Patient data		
						Sex/ age, y	Sample site	Sputum smear
HB002	■■□■■□■□□□□□■■■□■■■■■■■■■■■■■■■■■■■■■■□□□□□	ST683	SB0140	A1	4-11-4-7-5- 1-5-8-3-2	F/81	Sputum	+
HB008	■■□■■□■□□□□□■■■□■■■■■■■■■■■■■■■■■■■■■■□□□□□	ST683	SB0140	A1	3-10-4-5-5- 1-5-8-3-4	F/86	Sputum	–
HB010	■■□■■□■□□□□□■■■□■■■■■■■■■■■■■■■■■■■■■■□□□□□	ST683	SB0140	A1	4-3-4-7-5- 1-5-8-3-4	F/33	Sputum	+
HB011	■■□■■□■□□□□□■■■□■■■■■■■■■■■■■■■■□■■■■■□□□□□	ST680	SB0139	NA	4-3-4-7-3- 1-5-8-3-4	F/29	Sputum	+
HB012	■■□■■□■□□□□□■■■□■■■■■■■■■■■■■■■■□■■■■■□□□□□	ST680	SB0139	NA	4-10-4-7-4- 1-5-8-3-4	M/62	Sputum	+
HB006	■■□■■□■□□□□□■■■□■■■■■■■■■■■■■■■■■■□■■■□□□□□	ND	SB1186	NA	4-11-4-5-5- 2-5-9-4-4	M/60	Urine	NA
HB007	■■□■■□■□□□□□■■■□■■■■■■■■■■■■■■■■■■□□■■□□□□□	ND	SB1047	NA	4-10-3-7-7- 2-4-x-4-2	F/74	Neck abscess	NA
HB003	■■□■■□■□□□□□■■■□■■■■■■■■■■■■■■■■□□■■■■□□□□□	ST679	SB0142	D1	3-11-4-7-5- 1-6-8-3-4	M/37	Spinal disc aspirate	NA
HB009	■■□■■□■□□□□□■■■□■■■■■■■■■■■■■■■■■■□■□■□□□□□	ND	SB1187	NA	4-11-4-7-5- 2-5-10-4-4	F/80	Sputum	+
HB001	■■□■■■■■□■□■■■■□■■■■■■■■■■■■■■■■■■■■■■□□□□□	ST691	SB130	A2	2-11-3-7-5- 2-3-8-2-3	M/16	Sputum	+
HB005	■■□■■■■■□■□■■■■□■■■□□■■■■■■■■■■■■■■■■■□□□□□	ND	SB1185	NA	4-10-1-4-5- 2-6-11-7-4	M/68	Testis biopsy sample	NA

*VNTR, variable number tandem repeats; +, smear positive by auramine and Ziehl-Neelsen stains; −, smear negative by auramine and Ziehl-Neelsen stains; NA, not applicable; ND, not described; x, not determined. †Spoligotype designation as described in the SpolDB4 database (www.pasteur-guadeloupe.fr/tb). ‡Spoligotype assignment as described in the Mbovis.org database (www.mbovis.org). §Spoligotype assignment (*11*). ¶VNTR profile based on a 10-loci scheme for *M. bovis* strains from Ireland in this order: QUB1895, QUB11a, QUB11b, ETR-A, ETR-B, MIRU 24, MIRU 26, QUB 3232, QUB 3336, and QUB26 (*8*).

Six-locus VNTR (ETR-A–ETR-F) showed little variation. The predominant profile (5 strains) was 7-5-5-4*-3-3.1. All strains except CUH-HB005 had ETR A–F profiles, matching spoligotype SB0140 strains isolated from cattle in England and Wales (*12*). A 10-locus expanded panel derived from loci recently described for Irish zoonotic *M. bovis* strains (*8*) improved strain discrimination by producing different profiles for every isolate (Table 1). Hence, VNTR typing was able to split the clustered spoligotypes into individual profiles. Analysis of regions of difference confirmed that none of the strains were derived from *M. bovis* BCG.

## Conclusions

A recent outbreak report describing sputum-positive *M. bovis* disease transmitted by person-to-person contact (*6*) underlines the need for precise genetic markers of *M. bovis* to aid epidemiologic traceback. We studied strains of *M. bovis* isolated from humans in the Republic of Ireland, and we have defined an optimal set of markers using a combination of spoligotyping and VNTR. We detected a group of isolates (Table 2) of spoligotype SB0140, which is predominant in animal strains of *M. bovis* reported from the Republic of Ireland (51.8% of isolates) (*11*) and the United Kingdom (*1*). It forms the single largest group of *M. bovis* strains isolated from humans in the United Kingdom (30%) (*1*). This group was not reported in a recent series of *M. bovis* isolates from humans in Italy (*13*) or France (*14*). Indeed, none of the spoligotypes in our survey and these reports overlap. Predominant strains by spoligotype in animals and those infecting humans in the same country are known to be linked (*1**,**13**,**14*). We found 3 novel spoligotypes similar to SB0140 in a small group of patients, showing that a wider variety of strains infect humans than animals, as described in similar studies (*1**,**2**,**13*).

In our study, 81% of patients infected were >30 years of age (Table 2), comparable with findings of a previous survey of the southwest Ireland population (*5*). Primary infection of this group is likely to have been several decades before diagnosis, and our isolates probably represent reactivation of disease acquired earlier in life, effectively a record of past prevalence in animals. Spoligotype SB0140 strains were isolated from 2 patients who were >80 years of age, showing that the current predominance of SB0140 in animals (*11*) is therefore of long duration in Ireland, potentially going back 8 decades.

Molecular typing by insertion sequence (IS) *6110*–based restriction fragment length polymorphism (RFLP) is inadequate for *M. bovis* because of low copy numbers of IS*6110*. A combination of MIRU-VNTR and spoligotyping gives better discrimination than the RFLP method (*15*). Traditional 6-locus VNTR (ETR-A–ETR-F) has been described for typing of *M. tuberculosis* complex strains (*7*) including *M. bovis* (*2*), but an expanded panel with an additional 10 loci applied to our strains greatly improved discrimination and enabled individual identification of each isolate.

Seven of our 11 isolates were from sputum, and 6 were detected on direct smear with potential for transmission. A VNTR-typing scheme based on the loci established on *M. bovis* isolates from animals (*8*) would detect *M. bovis* clusters derived from foodborne outbreaks or horizontal transmission of disease between humans in Ireland. Our study provides ways to markedly improve the ability to identify and contact-trace future clusters of *M. bovis* infection in humans.
